# Green Synthesis of Silver Nanoparticles Using *Jasminum nudiflorum* Flower Extract and Their Antifungal and Antioxidant Activity

**DOI:** 10.3390/nano13182558

**Published:** 2023-09-14

**Authors:** Qian Yang, Juan Guo, Xiaofu Long, Chunyang Pan, Guoqin Liu, Jiantao Peng

**Affiliations:** Guizhou Key Laboratory for Tobacco Quality Research, College of Tobacco Science, Guizhou University, Huaxi District, Guiyang 550025, China

**Keywords:** green synthesis, AgNPs, *Jasminum nudiflorum*, antifungal activity, antioxidant activity

## Abstract

The synthesis of metal nanomaterials is a timely topic due to their widespread use in fields such as crop protection, the environment, medicine, and engineering. Green synthesis of nanoparticles, which uses plant extracts instead of industrial chemical agents to reduce metal ions, has been developed to decrease costs, reduce pollution, and improve environmental and human health safety. In this paper, silver nanoparticles (AgNPs) were synthesized from the flower extract of *Jasminum nudiflorum*. The green synthesized AgNPs were characterized by UV-Vis, FTIR, XRD, SEM, and other technologies. The antifungal activity of the prepared AgNPs against *Alternaria longipes* was tested using the plate method, the concentration dilution method, and other methods, and the antioxidant activity of the prepared AgNPs was evaluated by DPPH and hydroxyl free scavenging methods. The results showed that AgNPs synthesized from *J. nudiflorum* flower extract have a face-centered cubic structure (fcc), and the average grain size of the nanoparticles is 13 nm; they are also mainly spherical in shape. Additionally, the concentration of AgNPs (ranging from 16 to 128 μg/mL) significantly inhibited the mycelial growth of *A. longipes* in comparison to the control. The inhibitory rate gradually increased with increasing AgNP concentration, ranging from 70.64% to 79.60% at a concentration of 128 μg/mL. The minimum inhibitory concentration was observed at 32 μg/mL. AgNPs induced overaccumulation of MDA in *A. longipes*, resulting in cell membrane damage and nucleic acid leakage. Moreover, the AgNPs have significant antioxidant properties, which increase with increasing concentration. The clearance rate of DPPH was 25.46 ± 0.90% when the concentration of AgNPs was 8 μg/mL, and the clearance rate of the hydroxyl radical was 28.62 ± 0.59% when the concentration of AgNPs was 128 μg/mL. Thus, the flower extract from *J. nudiflorum* holds potential as an environmentally friendly and green alternative for the synthesis of AgNPs, which have antifungal and antioxidant potential.

## 1. Introduction

Nanotechnology is a branch of materials science and is considered to be one of the key technologies of the future [1]. The emerging field of nanotechnology research refers to the implementation of nanostructures or nanomaterials in the design, development, and application [2]. Nanomaterials refer to substances with a size of about one billionth of a meter and a length of approximately 100 nm [3,4]. These nanomaterials are classified as zero-dimensional, one-dimensional, two-dimensional, and three-dimensional nanomaterials [2,5]. In the context of nanotechnology, zero-dimensional nanoparticles are materials that possess a nanoscale dimension (below 100 nm) in all three dimensions of space [5]. Nanomaterials exhibit distinct properties, such as special surface area, volume, quantum size, and macroscopic tunneling effects, owing to their small particle size. Their unique optical, mechanical, catalytic, and biological properties make nanoparticles useful in various fields, such as biomedicine, industry, and agriculture [6,7,8].

Nanoparticles can be synthesized using physical, chemical, or biological methods. Physical methods encompass laser ablation, evaporation condensation, and mechanical ball milling [9,10,11,12]. On the other hand, chemical methods involve sol gel, microwave-assisted, and microemulsion techniques [8,13,14,15]. These physical and chemical methods commonly employ reducing agents, such as hydrogen peroxide, carbon monoxide, hydroxylamine hydrochloride, dimethylformamide, sodium borohydride, and hydrazine, for nanoparticle synthesis. However, it is important to note that these chemicals can be hazardous. In contrast, biological methods employ microorganisms or plants as a means to produce nanoparticles, utilizing natural components within plants as both reducing and capping agents. Biosynthetic methods offer several advantages, including simplicity, environmental friendliness, low cost, biocompatibility, scalability for large-scale production, and biodegradability [8,16].

Inorganic materials, particularly metal nanoparticles (NPs), have been found to possess antibacterial properties and exhibit greater stability under harsh conditions and high temperatures compared to organic materials [17]. AgNPs are one of the most commonly used nanomaterials because of their antimicrobial properties against a variety of pathogenic bacteria, and silver ions released from AgNPs are thought to play a crucial role in antibacterial activity [18,19]. The effects and toxicity of silver nanoparticles (AgNPs) on microorganisms, plants, and the environment have been extensively researched. These include their mode of action, transformation, transportation within the food chain, and their toxic effects on various plant systems. This also extends to their applications in biomedical and agricultural technology [20]. Studies have demonstrated that AgNPs enhance the germination rate of cabbage seeds, seedling growth, and yield. Furthermore, silver (Ag) does not bioaccumulate in edible tissues, thereby affirming the biosafety of AgNPs [21]. Xiang et al. observed that the life activities of crucian carp in both the clean water control group and the AgNP treatment group remained normal within a 96 h period, with no signs of restlessness or erratic swimming behavior [22]. AgNPs synthesized from petal extract of *Cydonia oblonga* exhibited an inhibitory zone diameter of 10 ± 0.80 mm against *Erwinia amovora*, a pathogenic bacterium that causes fire blight. Furthermore, AgNPs significantly reduced both the diameter and depth of the necrotic area of immature *Pyrus communis* fruits. In comparison, the necrotic zone diameter on fruits inoculated with pathogens alone was 32 ± 0.45 mm, almost eight times larger than that observed when bacterial and AgNPs suspensions were applied [23]. AgNPs also demonstrated antifungal potential. Green synthesis of AgNPs from leaves of *Pongamia glabra* resulted in a significant reduction in the weight of mycelium and spore count of the plant pathogen *Rhizopus stolonifera* [24]. AgNPs synthesized from rice leaves displayed antimicrobial activity against *Rhizoctonia solani*. At a concentration of 10 μg/mL, AgNPs exhibited maximum inhibition of 96.7% against TS-06, followed by 93% inhibition against TS-22. The minimum inhibitory concentration (MIC) values of AgNP on mycelium and sclerotia of *R. solani* were 5–10 and 15–20 μg/mL, respectively. AgNP treatment resulted in a significant decrease in percent disease incidence (PDI) values under in vitro conditions, and Ag NP at a concentration of 20 μg/mL completely inhibited the incidence of all *R. solani* isolates. [25]. AgNPs derived from wheat leaf extract demonstrated resistance against four fungi. The MIC of AgNPs against *Fusarium oxysporum* was found to be 41.7 μg/mL, while for *Aspergillus versicolor*, *A. niger*, and *A. brasiliensis*, it was determined to be 208.3 μg/mL [18]. Compared with the control, the green synthesis of AgNPs with 10.7 μg/mL Citrus limetta peel extract increased the content of protein and inhibited bacterial and yeast pathogens. AgNPs would damage the cell membrane integrity of bacteria and candida, cause changes in cell morphology, and lead to cell foaming and thick exudate deposition around cells [26].

Plant-based synthesis of silver nanoparticles has been achieved using a variety of plant materials, including rice leaves, wheat leaves, the leaves of Pongamia glabra, petals of *C. oblonga*, *Citrus limetta* peel, *Cleome viscosa*, and other plants [18,23,24,25,26,27]. Plant extracts abundant in phytoconstituents, such as alkaloids, flavonoids, and terpenoids, have the ability to reduce metal salts from Ag^+^ to Ag^0^, leading to the synthesis of AgNPs [28]. Due to the presence of diverse biomolecules in different plants, nanoparticles generated from distinct plant sources may demonstrate varying antimicrobial properties. Hence, it is imperative to explore new plant extract sources for nanoparticle synthesis to enhance the antimicrobial efficacy of the nanoparticles [17]. *Jasminum nudiflorum* Lindl, commonly cultivated as an ornamental plant and used in traditional medicine in China, is distinguished by the presence of dicyclic iridoid glycosides, pigments, flavonoids, and fatty acids in its flowers [29]. This study aimed to characterize the size and shape of green-synthesized silver nanoparticles (AgNPs) using an extract from *J. nudiflorum* flowers. Additionally, the study sought to assess the antibacterial activity of these green-synthesized AgNPs against *Alternaria longipes* and to evaluate their antioxidant activity. The research is distinguished by its validation of a more optimized synthesis scheme, which includes the volume ratio of plant extract to silver nitrate solution and reaction time. The AgNPs were synthesized from the *J. nudiflorum* flower extract without the need for catalysts or surfactants. The results indicate that these biosynthetic AgNPs can significantly inhibit *A. longipes*, the primary pathogen of tobacco leaf spot (tobacco brown spot), as well as exhibit antioxidant activity.

## 2. Materials and Methods

### 2.1. Preparation of Plant Materials and Extracts

The flowers of *J. nudiflorum* were collected from the campus of Guizhou University (106°66′ E, 26°45′ N) in Guiyang, Guizhou province, China. The flowers were washed with tap water, rinsed with ultrapure water to removes the dust particles, air-dried, and ground into fine powder with an electric grinder. The powder was passed through a 40-mesh sieve to remove large impurities. The extract was prepared by putting 1.5 g of powder together with 100 mL of ultrapure water in a 250 mL conical flask and heating it in a water bath at 80 °C for 60 min. The crude water extract was centrifuged at 10,000 rpm for 10 min and filtered with filter paper. Then, the extract was stored in a refrigerator at 4 °C for future research.

### 2.2. Preparation of AgNPs

**Figure 1:**
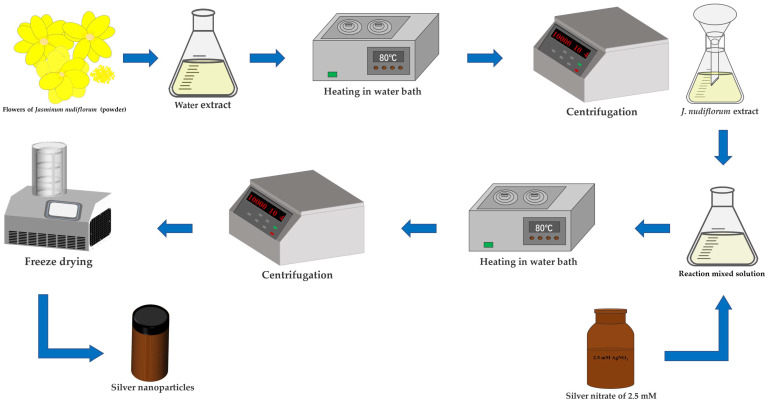
The process of green synthesis of AgNPs from *J. nudiflorum* flower extract.

The green-synthesis method is referred to by Mehata et al. [30]. To synthesize AgNPs, an orthogonal test was conducted to determine the optimal volume ratio of reaction mixture (A) and reaction time (B) (Table 1). Three different volume ratios of *J. nudiflorum* flower extract to a 2.5 mM AgNO_3_ (SCRC, Shanghai, China) solution were tested: 1 mL:1 mL for A1, 1.25 mL:1 mL for A2, and 1.5 mL:1 mL for A3. The reaction was carried out in a water bath at 80 °C for seven different durations: B1 for 1 h; B2 for 2 h; B3 for 3 h; B4 for 4 h; B5 for 5 h; B6 for 6 h; and B7 for 7 h, respectively. The mixtures were then centrifuged at 10,000 rpm for 10 min, and the precipitate was washed with ultrapure water to remove any unreacted reagents. Subsequently, the resulting AgNPs were freeze-dried into a powder and stored in a brown bottle. Figure 1 illustrates the process of synthesizing AgNPs from the flower extract of *J. nudiflorum.*

### 2.3. Characterization of AgNPs

To identify the structure, properties, and reaction mechanism of the synthesized nanoparticles, various characterization techniques were utilized [17]. The liquid mixture after reaction in the water bath was subjected to analysis via an Ultraviolet-Visible (UV-Vis) spectrophotometer (Analytic Jena AG, Specord 200 Plus, Jena, Germany). The silver nanoparticles (AgNPs), after freeze-drying, were examined using Fourier-transform infrared (FTIR) spectroscopy (Thermo Scientific Nicolet iS20, Waltham, MA, USA), X-ray diffraction (XRD) (Bruker D8 Advance, Mannheim, Germany) pattern analysis, and scanning electron microscopy (SEM) (ZEISS Sigma 300, Jena, Germany). The absorption spectra of the biosynthesized silver nanoparticles were measured using a UV-Vis spectrophotometer with a wavelength range of 280–700 nm. The FTIR spectroscopy was employed to identify the functional groups present in the silver nanoparticles within the range of 4000–400 cm^−1^. The XRD diffraction pattern was obtained using Cu Kα radiation at 40 kV and 40 mA. The SEM was utilized to assess the morphology and particle size distribution of the nanoparticles.

### 2.4. Antifungal Activity of AgNPs

The strain of *A. longipes* was isolated from tobacco leaves infected with brown spot disease in Meitan County, Guizhou Province and stored in the Guizhou Provincial Key Laboratory for Tobacco Quality Research.

#### 2.4.1. Effect of AgNPs on *A. longipes* Mycelium

The effect of AgNPs on the mycelium of *A. longipes* was evaluated by inoculating 6 mm diameter pathogenic bacteria cake in a PCA medium containing different concentrations of nanoparticles, taking 0 μg/mL nanoparticles as the control.

The effect of silver nanoparticles on *A. longipes* mycelium was evaluated by inoculating 6 mm diameter pathogenic cake in a PCA medium containing different concentrations of AgNPs, with 0 μg/mL NPs serving as the control. The mycelium dimension was measured at 3, 6, and 9 days, and the inhibition rates were calculated. Each treatment was replicated three times [22]. The inhibition rate was calculated as follows:

$$\begin{array}{c} \mathrm{Inhibition}\;\mathrm{rate}\;\left(\%\right)\\ =\frac{\left(Control\;group\;colony\;growth\;diameter-Treatment\;group\;colony\;growth\;diameter\right)}{Control\;group\;colony\;growth\;diameter}\times 100 \end{array}$$

#### 2.4.2. Effect of AgNPs on Membrane Permeability and Malondialdehyde Content of *A. longipes*

*A. longipes* was cultured in PDB medium following inoculation and incubated in a rotating shaker at 150 rpm and 28 °C for a duration of 10 days. The mycelia were collected by centrifugation at 8000× *g* for 5 min using a filter centrifuge tube. Subsequently, 0.2 g of mycelium was placed in a PDB culture medium containing different concentrations of nanoparticles. The mixture was cultured in a rotating shaker at 150 rpm and 28 °C for 0, 2, 4, and 8 h to assess the selected parameters. This experimental process was repeated three times [31].

To determine cell membrane permeability, the mycelia were removed through centrifugation at 8000× *g* for 15 min, and the resulting supernatant was collected for subsequent measurement of cell leakage. Nucleic acid leakage was quantified at 260 nm using a spectrophotometer [31]. The formula for the quantity of DNA was as follows [32]:

$$\mathrm{Quantity}\;\mathrm{of}\;\mathrm{DNA}\;\left(\frac{\mu g}{\mathrm{mL}}\right)=\mathrm{OD}\;\mathrm{at}\;260\;\mathrm{nm}\times 50$$

The measurement of MDA content involved adding 0.2 g of mycelium to 2 mL of a 10% trichloroacetic acid (TCA) solution, which was then homogenized by grinding. After centrifuging at 8000× *g* and 4 °C for 15 min, the supernatant was collected. Subsequently, 1 mL of the supernatant was mixed with an equal volume of a 0.6% thiobarbituric acid (TBA) solution. The resulting mixture was boiled for 30 min, rapidly cooled to room temperature using tap water, and measured at 450 nm, 532 nm, and 600 nm. The MDA content was expressed as μmol/g [31].

$$MDA\;\left(\mu \mathrm{mol}.g^{-1}\right)=\frac{\left[6.452\times \left(A_{532}-A_{600}\right)-0.559\times A_{450}\right]\times V_{t}}{V_{s}\times m}$$

In this formula, *Vt* represents the total volume (mL) of the extract, *Vs* represents the volume (mL) of the extract for measurement, and *m* represents the sample weight (m).

#### 2.4.3. Determining the Minimum Antifungal Concentration of AgNPs

The minimum inhibitory concentration (MIC) of silver nanoparticles was deter-mined by visually observing the growth of fungi in continuously diluted suspensions of nanoparticles. Initially, PDB and nanoparticles were mixed in the first conical flask and subsequently used to dilute the ensuing conical flasks. Next, a spore suspension containing 2 × 10^5^ spores/mL was inoculated into each conical flask, and the cultures were incubated at 28 °C for a period of 3 days. Visual observation of fungal growth was con-ducted to ascertain the value of the MIC. This experiment was repeated three times [31].

### 2.5. Antioxidant Activity of AgNPs

The antioxidant activity of silver nanoparticles was evaluated using two different assays. The first assay, based on the 1,1-diphenyl-2-trinitrophenylhydrazine (DPPH) radical scavenging method, was conducted with a slight modification of the procedure outlined by Dridi et al. [33]. In this assay, a 1 mL sample solution was mixed with an equal volume (1 mL) of DPPH solution. After vigorous shaking, the mixture was allowed to react in darkness for 30 min. The absorbance of the resulting solution was measured at 517 nm, and the percentage of inhibition was then calculated using the following formula:

$$\mathrm{DPPH}\;\mathrm{clearance}\;\mathrm{rate}\;\left(\%\right)=\frac{\left(A_{0}-\left(A_{i}-A_{j}\right)\right)}{A_{0}}\times 100$$

In this formula, *A*_0_ represents the absorbance of a solution containing 1 mL anhydrous ethanol and 2 mL DPPH, *A_i_* represents the absorbance of a solution containing 1 mL sample and 2 mL DPPH, and *A_j_* represents the absorbance of a solution containing 1 mL sample and 2 mL anhydrous ethanol.

For the second assay, the hydroxyl radical scavenging activity of the AgNPs was determined following the procedure described by Ansar et al. [34]. A reaction mixture consisting of approximately 3 mL was prepared by adding 1 mM salicylic acid, 9 mM ferrous sulfate, and 1 mL hydrogen peroxide to 2 mL of synthesized AgNPs. The mixture was then subjected to a reaction at 60 °C in a water bath for 37 min. After incubation, the absorbance of the resulting solution was measured at 510 nm. The percentage of hydroxyl radical scavenging activity was calculated using the following formula:

$$\mathrm{Hydroxyl}\;\mathrm{radical}\;\mathrm{scavenging}\;\mathrm{rate}\;\left(\%\right)=\frac{\left(A_{0}-\left(A_{i}-A_{j}\right)\right)}{A_{0}}\times 100$$

In this formula, *A*_0_ represents the absorbance of a solution containing 1 mL salicylic acid, 1 mL ferrous sulfate, 1 mL hydrogen peroxide, and 2 mL ultrapure water; *A_i_* represents the absorbance of a solution containing 1 mL salicylic acid, 1 mL ferrous sulfate, 1 mL hydrogen peroxide, and 2 mL sample; and *A_j_* represents the absorbance of a solution containing 1 mL salicylic acid, 1 mL ferrous sulfate, 1 mL ultrapure water, and 2 mL sample.

### 2.6. Data Processing

The data were presented as the standard error of the mean, based on three replicates. To compare the means between treatments, analysis of variance (ANOVA) and least significant difference (LSD) tests were conducted using SPSS 26.0 software. A significance level of *p* < 0.05 was used to determine statistical significance.

## 3. Results and Discussion

### 3.1. Production of Silver Nanoparticles

**Figure 2:**
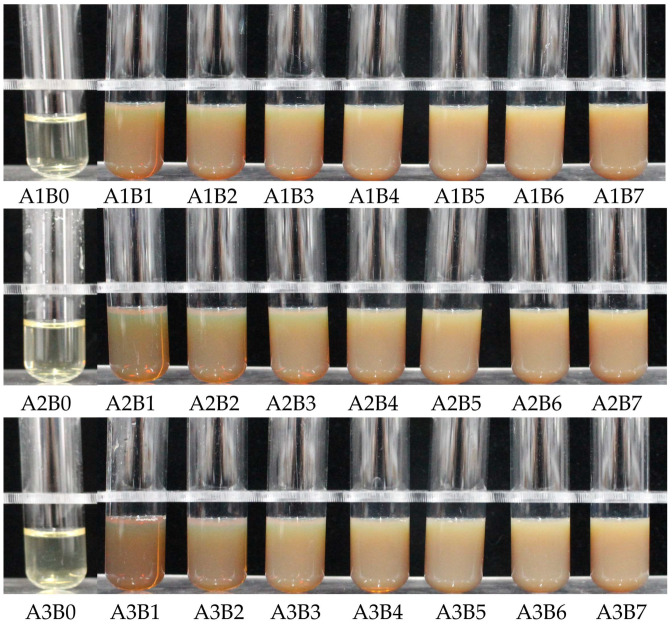
Photographs of the process change of AgNPs synthesized using the flower extract of *J. nudiflorum*. A1–A3 represents the volume ratios of the extract to the AgNO_3_ solution of 1 mL:1 mL, 1.25 mL:1 mL, and 1.5 mL:1 mL, respectively; B0–B7 represents reaction times of 0 h, 1 h, 2 h, 3 h, 4 h, 5 h, 6 h, and 7 h, respectively.

**Figure 3:**
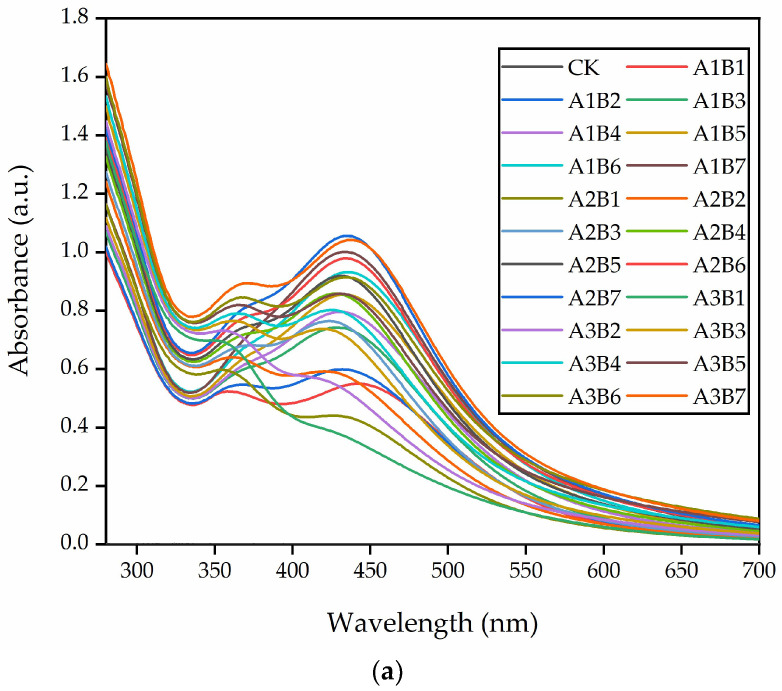

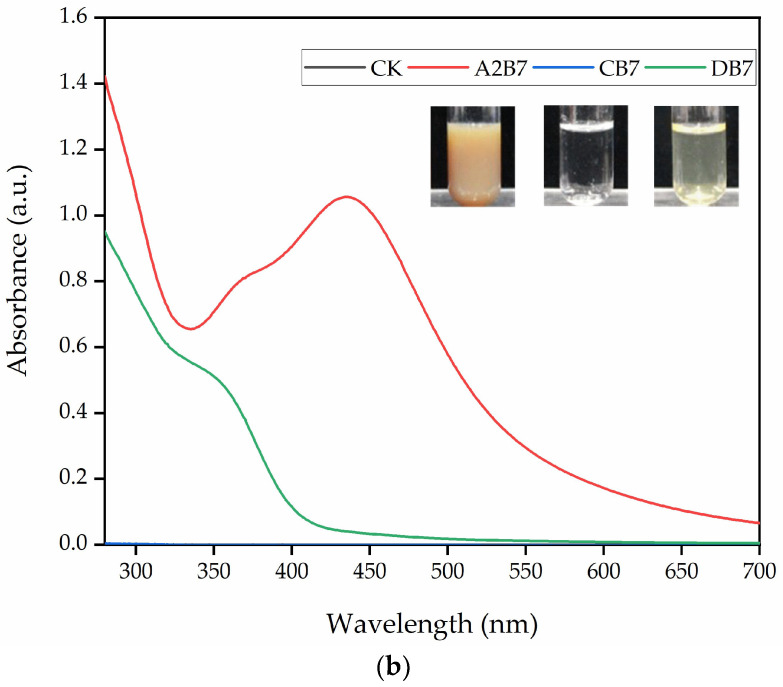
UV-Vis absorption spectra of AgNPs green-synthesized by *J. nudiflorum* flower extract. (**a**) The UV-Vis absorption spectra of AgNPs synthesized using the flower extract. CK represents ultrapure water; A1–A3 represents the volume ratios of the extract to the AgNO_3_ solution of 1 mL:1 mL, 1.25 mL:1 mL, and 1.5 mL:1 mL, respectively; B0–B7 represents reaction times of 0 h, 1 h, 2 h, 3 h, 4 h, 5 h, 6 h, and 7 h, respectively. (**b**) The UV-Vis absorption spectra of A2B7; CB7, which involved a ratio of 1.25 mL of ultrapure water to 1 mL of AgNO_3_, and heated for 7 h; and DB7, which involved a ratio of 1.25 mL of the flower extraction solution to 1 mL of ultrapure water and was also heated for 7 h.

To determine the optimal synthesis ratios of the flower extract of *J. nudiflorum* and AgNO_3_ solution and reaction times for synthesizing AgNPs, three different ratios of plant extracts to AgNO_3_ solution (1 mL:1 mL, 1.25 mL:1 mL, and 1.5 mL:1 mL) and seven different reaction durations (1 h, 2 h, 3 h, 4 h, 5 h, 6 h, and 7 h) were prepared, resulting in a total of 21 experimental conditions. The reaction between the flower extracts of *J. nudiflorum* and AgNO_3_ resulted in a visible color transition from yellowish to reddish-brown, indicating the successful green synthesis of AgNPs (Figure 2). This color alteration was attributed to the surface plasmon resonance (SPR) of silver ions, which were reduced to stable AgNPs by active molecules in the extract [30,35]. At 1 h, the red-brown color was clearly observed in A1B1, A2B1, and A3B1, indicating the initiation of AgNP synthesis. However, as the reaction time was extended to 2–7 h, no significant visual changes were observed among A1, A2, and A3. Nevertheless, over time, the color of the reaction mixture with the same ratio of plant extract to AgNO_3_ solution became slightly lighter, and the solution became gradually denser (Figure 2). Additionally, the 21 treated reaction mixtures were characterized using UV-Vis spectroscopy (Figure 3a). At 1 h, the reddish-brown color of A1B1 was lighter and the solution was thicker compared to A2B1 and A3B1 (Figure 2). The UV-Vis spectrum showed an increase in absorbance for A1B1(Figure 3a), indicating the presence of a greater number of silver nanoparticles [36]. As time progressed, each reaction solution became denser and the absorbance increased (Figure 2 and Figure 3a), suggesting that more AgNPs were synthesized.

### 3.2. Analysis of UV-Vis Absorption Spectra of Green-Synthesized AgNPs

The UV-Vis spectrum of the AgNP liquid, as depicted in Figure 2, revealed an absorption peak within the 280~700 nm range, with a prominent peak at approximately 435 nm when compared to the control (CK, ultrapure water) as shown in Figure 3a. The absorption peak around 330 nm might be attributed to the plant extract (Figure 3b). This UV-Vis analysis confirmed the formation of AgNPs. Typically, the UV-Vis spectrum of AgNPs presents a strong visible light band around 435 nm (Figure 3a), and the 350–550 nm range is identified as the typical SPR absorption band of AgNPs. The absorption peak for spherical AgNPs is usually between 410 and 450 nm [37,38]. It was observed that with an increasing reaction time (1–7 h), and a volume ratio of *J. nudiflorum* flower extract to AgNO_3_ solution at 1 mL:1 mL, AgNPs exhibited a blue shift, indicating the formation of smaller AgNPs [30,39], and an increase in absorption spectral intensity, suggesting that more AgNPs were synthesized [30,40]. When the volume ratio of flower extract to AgNO_3_ solution was increased to 1.25 mL:1 mL and 1.5 mL:1 mL, a redshift phenomenon was observed in AgNPs, and the absorption spectral intensity increased with extended reaction time (1–7 h). In the ultraviolet absorption spectrum, the absorption peak of A2B7 (435 nm, 1.0561) is positioned between A1B7 (433 nm, 1.0009) and A3B7 (438 nm, 1.0418), with A2B7 exhibiting a higher peak. As a result, AgNPs synthesized under A2B7 condition were selected for further investigation.

In this study, the AgNPs synthesized in test A2B7 by the flower extract of *J. nudiflorum* exhibited a maximum absorption peak at 435 nm, corresponding to surface plasmon resonance [41]. By comparing the UV-Vis spectra of the flower extract and AgNO_3_ (Figure 3b), at 435 nm, there was no absorption peak in the flower extract of *J. nudiflorum* and AgNO_3_ solution, the formation of AgNPs was confirmed (results that are similar to the study of Takcı et al. [28]), and there was no absorption peak in the plant extract at the maximum absorption peak of AgNPs. Further experiments were then conducted on AgNPs synthesized under A2B7 conditions.

### 3.3. Fourier Infrared Spectroscopy of Green-Synthesized AgNPs

**Figure 4:**
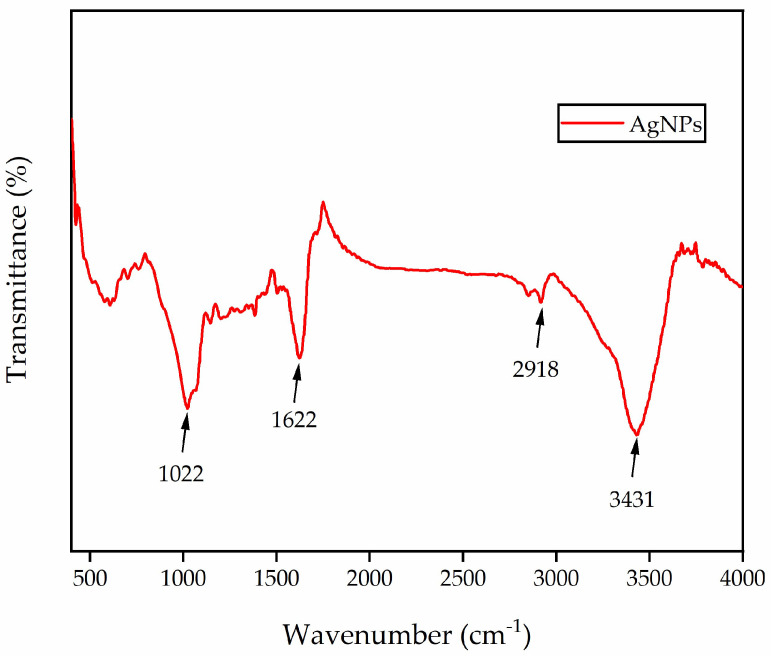
FTIR spectra of AgNPs synthesized from the flower extracts of *J. nudiflorum*.

The FTIR analysis was employed to identify the functional groups potentially involved in the synthesis of AgNPs. In the case of the green synthesis of NPs, plant extracts are utilized as both reducing and stabilizing agents, presumably due to the presence of various metabolites, including flavonoids, alkaloids, amino acids, proteins, vitamins, and enzymes [8,42,43]. The FTIR spectra of the synthetic AgNPs derived from *J. nudiflorum* flower are presented, which display prominent peaks at 3431 cm^−1^, 2918 cm^−1^, 1622 cm^−1^, and 1022 cm^−1^ (Figure 4). These peaks can be attributed to the vibrations of O-H, C-H, C=C stretching, and C-O stretching, respectively. Notably, the substantial absorption peaks observed at 1029 cm^−1^, 2922 cm^−1^, and 3414 cm^−1^ primarily correspond to the polyphenols extracted from the plants [28]. Additionally, peaks at 3795 cm^−1^, 3452 cm^−1^, 3439 cm^−1^, 3430 cm^−1^,3422 cm^−1^, 3418 cm^−1^, 3360 cm^−1^, and 3255 cm^−1^ can be ascribed to the stretching vibrations of the O-H or N-H groups present in alcohol or phenol compounds that bind to the surface of AgNPs [44,45,46,47,48].

Moreover, peaks at 2930 cm^−1^, 2923 cm^−1^, 2922 cm^−1^,2921 cm^−1^, 2900 cm^−1^, and 2880 cm^−1^ are associated with the C-H stretching vibrations of alkanes [23,28,35,46,47]. The presence of aldehyde or ketone functional groups (C=O) from flavonoid and tannin derivatives can be inferred from the peaks at 1617 cm^−1^,1626 cm^−1^, 1650 cm^−1^, and 1653 cm^−1^ [44,47,49]. While the peak at 1633 cm^−1^ and 1631 cm^−1^ signifies C=C stretching vibrations of the hydrocarbon portion of biological double-bond molecules, the band at 1052 cm^−1^ and 1031 cm^−1^ may be attributed to C-O stretching [35,46,50]. And the peaks at 1607 cm^−1^ and 1396 cm^−1^ correspond to the asymmetric stretching of NO_2_ [50]. Flavonoids, monoterpenes, iridoids, mites, and glycosides constitute the major compounds in the *Oleaceae* Hoffmans. & Link family of *J. nudiflorum* [51].

### 3.4. Analysis of Green-Synthesized AgNPs through X-ray Spectroscopy

**Figure 5:**
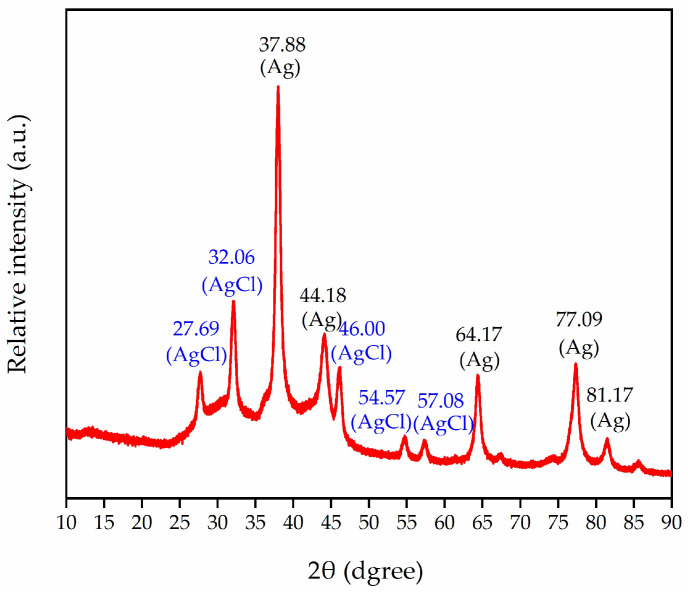
XRD patterns of AgNPs synthesized from the flower extract of *J. nudiflorum*.

To ascertain the crystallinity of AgNPs, powder XRD analysis was conducted on the AgNPs. The XRD spectrum of AgNPs green-synthesized using the flower extract of *J. nudiflorum* demonstrates that the diffraction peaks align with the standard silver planes (PDF: 04-003-7118) at angles of 37.88°, 44.18°, 64.17°, 77.09°, and 81.17°, respectively, corresponding to the (111), (200), (220), (311), and (222) planes (Figure 5). This confirms the face-centered cubic (fcc) crystal structure of AgNPs. Compared with the standard silver characteristic peaks 37.98°, 44.14°, 64.20°, 77.09°, and 81.21°, there are slight differences in peak positions, which may be related to the residual plant extract on the surface of the synthesized silver nanoparticles [52]. In addition, 27.69°, 32.06°, 46.00°, 54.57°, and 57.08°corresponded to the (111), (200), (220), (311), and (222) planes of the standard AgClNPs (PDF:97-006-4734), respectively (Figure 5). This was similar to the XRD spectra of Kohan Baghkheirati [53] and Kumkoon et al. [54]. The formation of AgClNPs might be the result of the reaction of Ag in AgNO_3_ with the chemical compound Cl in the plant extract [54]. Additionally, the XRD spectrum showcased additional unmatched peaks, possibly attributable to the crystallization of bio-organic compounds on the surface of AgNPs. The noise is related to the phytochemical components in the possible extract of the impurity peak [24,49,54,55]. The size of AgNPs was calculated using Scherrer’s formula: $D=\frac{K\times \lambda }{\beta \times cos\theta }$. The size of the green-synthesized silver nanoparticles from the flower extract of *J. nudiflorum* was about 13 nm.

### 3.5. Analysis of the Morphology and Size Range of Green-Synthesized AgNPs Using Scanning Electron Microscopy

**Figure 6:**
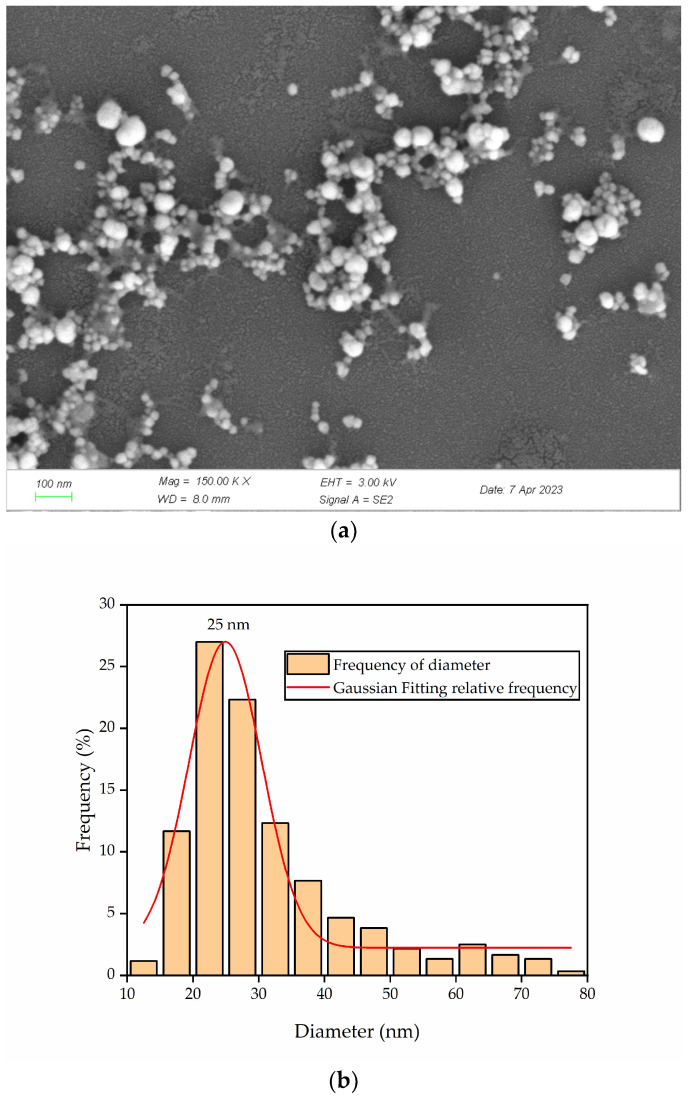
SEM of AgNPs synthesized using the flower extract of *J. nudiflorum*. (**a**) The SEM of AgNPs; (**b**) the particle size distribution of AgNPs.

The morphology and size of AgNPs synthesized from *J. nudiflorum* flower were examined using SEM images. The images revealed that the synthesized AgNPs were predominantly spherical in shape and exhibited good dispersion (Figure 6). The size of the synthesized AgNPs was measured using Nano Measurer, yielding a range of 11 to 78 nm, with an average size of approximately 25 nm (Figure 6b). Furthermore, these AgNPs displayed a predominantly spherical shape, which corresponds well with the characteristic SPR peaks observed in the UV-Vis spectrum [28]. It is worth noting that the green-synthesis method resulted in some agglomeration of the nanoparticles, as high surface activity and the absence of coordinating atoms on their surface tend to cause their aggregation [5].

### 3.6. Inhibitory Effect of Green-Synthesized AgNPs on A. longipes

**Figure 7:**
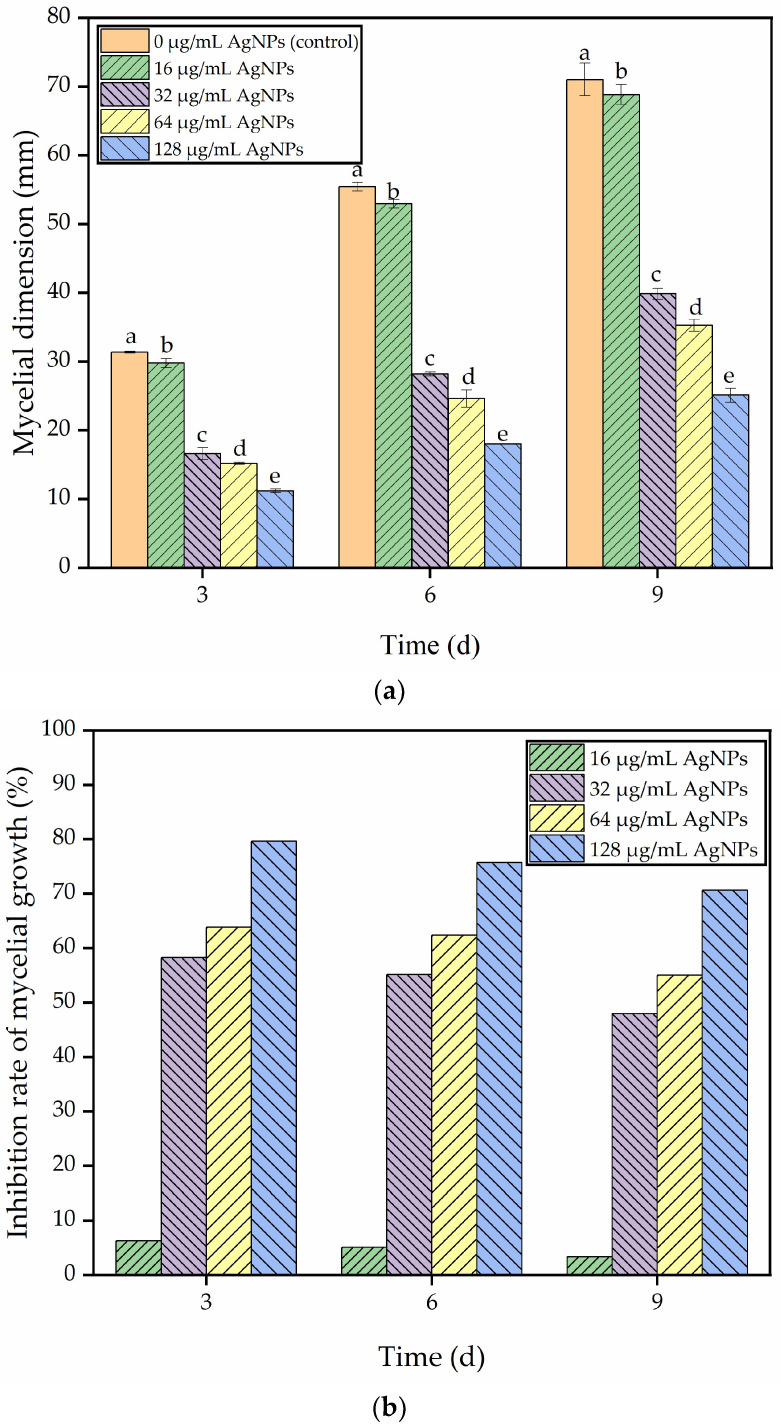

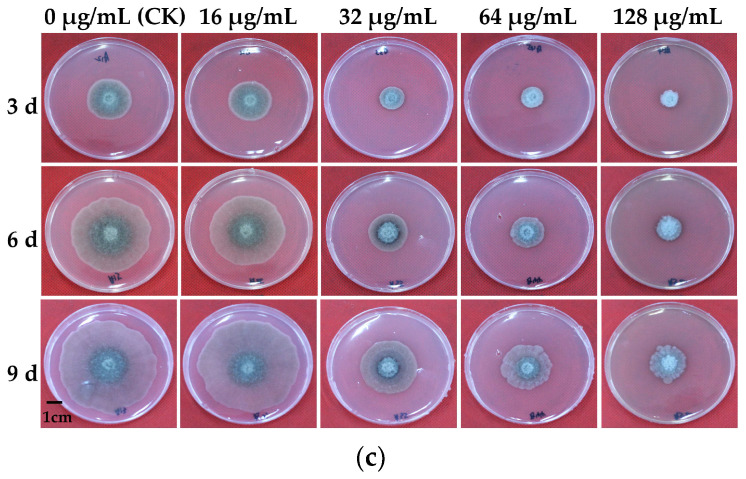
Effect of green synthesis of AgNPs from the flower extract of *J. nudiflorum* on *A. longipes*. (**a**) The effect of using green synthetic AgNPs on the mycelium dimension of *A. longipes* cultured in PCA medium for 3, 6, and 9 days. (**b**) The inhibition rate of mycelial growth of *A. Longipes* cultured in PCA medium containing AgNPs for 3, 6, and 9 days. (**c**) Images of mycelial growth of *A. Longipes* on PCA medium containing AgNPs after 3, 6, and 9 days of incubation. Values labeled with different letters between the control and AgNP treatment at the same time point indicate significant differences according to the LSD test (*p*  <  0.05).

The effect of AgNPs on the growth of *A. longipes* mycelium was investigated. Mycelium dimension was measured on days 3, 6, and 9 and compared to a control group (0 μg/mL AgNPs). The results showed that the growth of *A. longipes* mycelium was significantly inhibited by AgNPs in concentrations ranging from 16 to 128 μg/mL. The inhibition rate increased as the concentration of AgNPs increased, with ranges of 6.25–79.60%, 5.06–75.72%, and 3.33–70.64% observed at 3, 6, and 9 days, respectively (Figure 7a and Figure 7b). This finding supports the study by Malandrakis et al. [56], which demonstrated the inhibitory effect of NPs on the in vitro mycelial growth of fungal strains.

To further investigate the impact of AgNPs on the culture of *A. longipes*, the strain was cultured on PCA medium with varying concentrations of AgNPs, and the colony morphology was observed throughout the cultivation period (Figure 7c). The results showed that the radial growth of *A. longipes* treated with AgNPs was slower compared to the control group, and the growth rate decreased with increasing concentrations of AgNPs. Similar results were found by Xiang et al. [22] in their study on the inhibitory effect of AgNPs on the growth of *Alternaria alternata*. However, Xiang et al. [22] noted that the inhibitory effect was more prominent at lower concentrations of AgNPs, which could be attributed to variations in strain sensitivity and the smaller particle size of their AgNPs. When the particle size is smaller, nanoparticles can easily penetrate the cell wall of microorganisms and enhance their absorption by microbial cells [57]. The presence of nanoscale micropores in biofilms, such as cell membranes (0.4–1 nm) and nuclear membranes (50–70 nm), as well as the cell wall micropores (5–20 nm), facilitate the direct entry of metal-based nanoparticles into cells and even organelles. Moreover, smaller particle sizes of metal-based nanoparticles promote their accumulation and potential toxicity in organisms [58]. The presence of negative surface charges on nanoparticles, which are closely associated with bacterial cell wall charges, can also reduce efficacy by causing repulsion and hindering absorption by bacteria [59]. Based on these findings, the concentration of 128 μg/mL AgNPs was selected to further investigate its antifungal mechanism.

### 3.7. Assessment of Cell Membrane Permeability and Malondialdehyde Content

**Figure 8:**
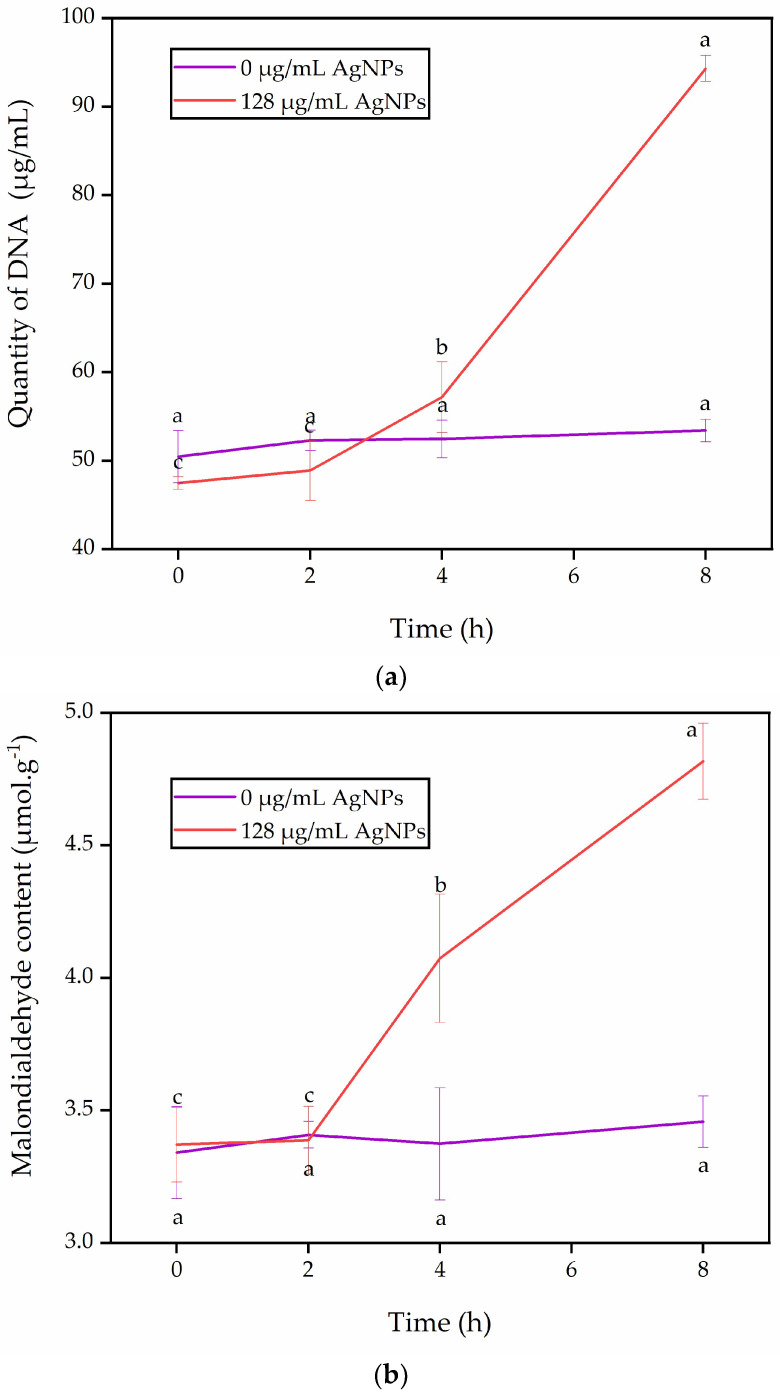
The effect of green-synthesized AgNPs on cell membrane permeability and malondialdehyde content of *A. longipes*. (**a**) Effect of green synthetic AgNPs on nucleic acid leakage of *A. longipes*; (**b**) the effect of green synthetic silver nanoparticles on malondialdehyde content of *A. longipes*. Values labeled with different letters at different time points of the same treatment were significantly different according to the LSD test at *p*  <  0.05.

Cell membrane permeability serves as a critical indicator of cell membrane impairment. AgNP treatment resulted in nucleic acid leakage in *A. longipes*. When *A. longipes* was treated with 0 μg/mL AgNPs, there was no notable change in nucleic acid leakage. However, nucleic acid leakage was detected after 4 h in *A. longipes* treated with 128 μg/mL AgNPs, and the leakage increased gradually with longer incubation times. After incubation with 128 μg/mL AgNPs for 8 h, the quantity of DNA 94.32 ± 1.48 μg/mL was significantly higher than the measured value of 47.48 ± 0.72 μg/mL at 0 h (Figure 8a). Xiang et al. [22]. observed that AgNP treatment disrupted the cell membrane permeability of *A. alternata*.

The MDA content showed no significant change in *A. longipes* treated with 0 μg/mL AgNPs over time. However, there was a significant change in the MDA content of *A. longipes* treated with 128 μg/mL AgNPs. After incubation for 8 h, the MDA content in *A. longipes* treated with 128 μg/mL AgNPs was 4.82 ± 0.14 μmol/g, which was significantly higher than 3.37 ± 0.17 μmol/g at 0 h (Figure 8b). This indicates that AgNPs induced lipid peroxidation in *A. longipes*. Excessive accumulation of MDA alters the structure and functionality of biomolecules on the cell membrane, including lipids, proteins, and nucleic acids, ultimately disrupting cell metabolism and potentially leading to cell death [60]. Hirpara et al. [61] discovered that the antifungal effect of green-synthesized AgNPs on MIC at day 3 involved mycelial cell membrane leakage (sugars and proteins), lipid peroxidation, inhibition of respiratory chain dehydrogenase activity, and disruption of the mycelium nucleus disc structure, resulting in the death of the plant pathogen. Furthermore, Zhu et al. [31] found that ZnO NPs induced excessive MDA accumulation in *A. alternata*, causing cell membrane damage and resulting in protein and nucleic acid leakage. Therefore, we propose that the green-synthesized AgNPs from the flower extract of *J. nudiflorum* inhibit the activity of *A. longipes* by inducing the excessive accumulation of MDA, which leads to increased membrane permeability. This mechanism may contribute to the antifungal properties of AgNPs.

### 3.8. Minimum Inhibitory Concentration of AgNPs

**Figure 9:**
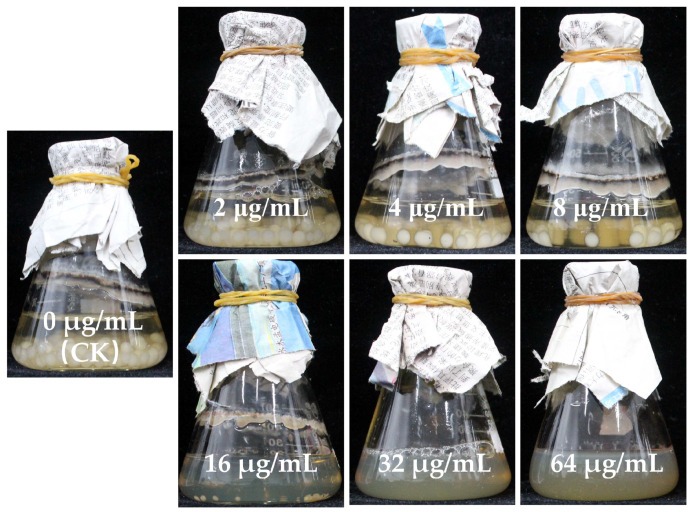
The inhibition of green-synthetic AgNPs on *A. longipes* in PDB medium, incubated for 3 days.

The minimum inhibitory concentration (MIC), which denotes the concentration at which *A. longipes* is completely inhibited, was evaluated. As demonstrated (Figure 9), the MIC value for AgNPs synthesized from spring green was 32 μg/mL. It is important to note that there existed a disparity in the inhibitory concentration of AgNPs against spores and mycelium, a finding aligned with Malandrakis et al.’s [56] study. Their research indicated that AgNPs, CuNPs, CuONPs, and ZnO_2_NPs generally exhibit toxicity levels towards spores that are 10 to 100 times higher than that to mycelium. These results suggest that AgNPs synthesized from the flower extracts of *J. nudiflorum* possess antifungal potential.

### 3.9. Antioxidant Capacity of Green Synthetic AgNPs

**Figure 10:**
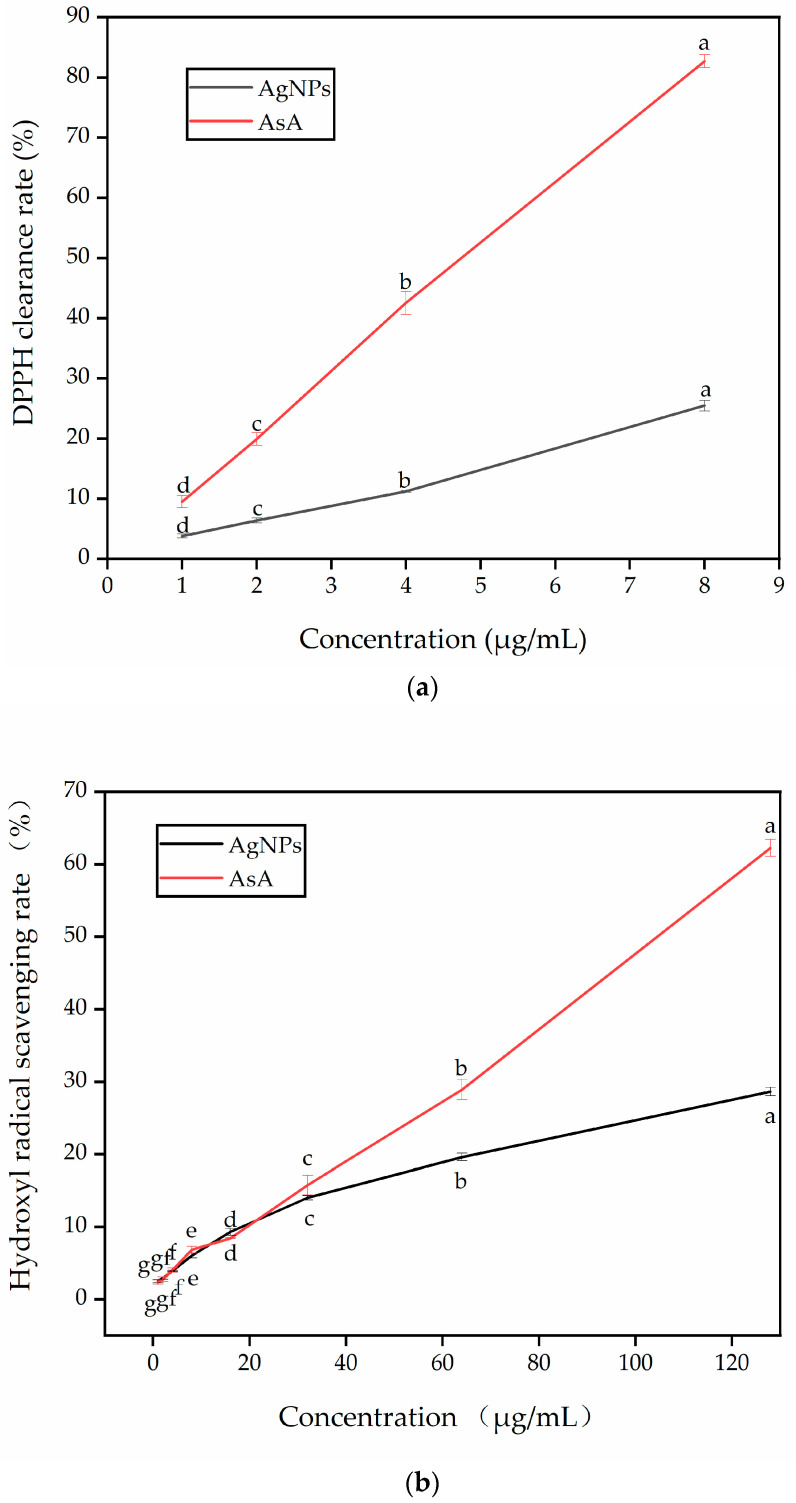
Antioxidant capacity of green synthesis AgNPs from the flower extract of *J. nudiflorum*. (**a**) The scavenging rate of AgNPs to DPPH, and AsA is the standard control; (**b**) the scavenging rate of AgNPs to hydroxyl radical, and AsA is the standard control. The same antioxidant is represented by the values labeled with different letters between different concentrations, and there is significant difference according to the LSD test at *p* < 0.05.

The antioxidant capacity of AgNPs was assessed through DPPH and hydroxyl radical scavenging experiments. Ascorbic acid (AsA) served as the standard antioxidant. The scavenging capacity of the sample for free radicals increased with higher concentrations of AgNPs (1–128 μg/mL), indicating its antioxidant properties (Figure 10). Consistent with the findings of Padalia et al. [41], the antioxidant activity also increased with increasing concentration. The DPPH (Figure 10a) and hydroxyl radical scavenging activities (Figure 10b) of AgNPs ranged from 3.80 ± 0.32% to 25.46 ± 0.90% and from 2.50 ± 0.22% to 28.62 ± 0.59%, respectively. The IC50 values for DPPH and hydroxyl radical scavenging with AgNPs were 16.07 and 222.53, respectively. Correspondingly, the IC50 values for AsA in DPPH and hydroxyl radical scavenging were 4.83 and 104.42, respectively. The scavenging potential of AgNPs was relatively lower compared to the significant antioxidant activity of the standard AgNPs, as supported by Ansar et al. [33]. Thus, the synthesized AgNPs demonstrated potential antioxidant properties, as evidenced by the aforementioned experiments.

## 4. Conclusions

This study presents a straightforward and eco-friendly approach to synthesizing AgNPs by employing *J. nudiflorum* flower extract for green synthesis. The utilization of *J. nudiflorum* flower extract serves dual purposes as both a reducing agent and a capping agent, thereby facilitating the formation of stable AgNPs. The UV-Vis spectrum peak, occurring at approximately 435 nm, and the X-ray diffraction patterns, specifically the 2θ peaks at 37.88°, 44.18°, 64.17°, 77.09°, and 81.17°, confirm the face-centered cubic (fcc) crystal structure of the AgNPs, and the particle size is about 13 nm. SEM examinations indicate that the particles exhibit predominantly spherical morphology, and FTIR spectroscopy analysis reveals prominent O-H, C-H, C=O, and C-O stretching vibrations. Furthermore, the synthesis of AgNPs exhibits notable antifungal effects, exhibiting an antifungal rate of 79.60% at 3 days, 75.72% at 6 days, and 70.64% at 9 days for AgNPs at a concentration of 128 g/mL. The minimum inhibitory concentration is determined to be 32 μg/mL, which induces an increase in MDA levels, damage to the cell membrane, and leakage of nucleic acids in *A. longipes*. Moreover, the green synthesized AgNPs show considerable antioxidant activity, with DPPH inhibition rates ranging from 3.80 ± 0.32% to 25.46 ± 0.90%, and hydroxyl radical scavenging capacities ranging from 2.50 ± 0.22% to 28.62 ± 0.59%. It seems that the green synthesis of AgNPs from the flower extract of *J. nudiflorum* provides a feasible alternative method for chemical synthesis, and the green-synthesized AgNPs has antifungal and antioxidant potential and is expected to be developed into an antifungal agent and an antioxidant.

## Figures and Tables

**Table 1:** Orthogonal experiment for determination of reaction mixture volume ratio (A) and reaction time (B) in the synthesis of AgNPs.

Factor A (Volume Ratio of *J. nudiflorum* Flower Extract to AgNO_3_ Solution)	Factor B (Reaction Time)						
	B1 (1 h)	B2 (2 h)	B3 (3 h)	B4 (4 h)	B5 (5 h)	B6 (6 h)	B7 (7 h)
A1 (1 mL:1 mL)	A1B1	A1B2	A1B3	A1B4	A1B5	A1B6	A1B7
A2 (1.25 mL:1 mL)	A2B1	A2B2	A2B3	A2B4	A2B5	A2B6	A2B7
A3 (1.5 mL:1 mL)	A3B1	A3B2	A3B3	A3B4	A3B5	A3B6	A3B7

## Data Availability

Data produced in this study can be made available upon reasonable request.
